# In Vitro and In Vivo Evaluation of Vancomycin-Loaded PMMA Cement in Combination with Ultrasound and Microbubbles-Mediated Ultrasound

**DOI:** 10.1155/2015/309739

**Published:** 2015-01-06

**Authors:** Tiao Lin, Xun-Zi Cai, Ming-Min Shi, Zhi-Min Ying, Bin Hu, Chen-He Zhou, Wei Wang, Zhong-Li Shi, Shi-Gui Yan

**Affiliations:** Department of Orthopaedic Surgery, Second Affiliated Hospital, School of Medicine, Zhejiang University, 88 Jiefang Road, Hangzhou, Zhejiang 310009, China

## Abstract

Ultrasound (US) has been used to increase elution of antibiotic from an antibiotic-loaded poly(methyl methacrylate) (PMMA) bone cement (ALBC). We aimed to further investigate whether microbubbles-mediated US (US + MB) facilitate elution of vancomycin (VAN) from cylindrical specimens and enhance the activity of the eluted antibiotic against* Staphylococcus aureus* (*S. aureus*) in vitro. The study groups comprised cylindrical bone cement fabricated with VAN (VAN), ALBC using US (VAN + US), and ALBC using MB-mediated US (VAN + US + MB). We also carried out an in vivo study involving the activity of VAN from cylindrical cement implanted in tibiae of New Zealand white rabbits inoculated with* S. aureus*. We found that (1) in vitro, elution from VAN + US + MB cylinders was significantly higher than from either the VAN or VAN + US specimens; (2) the activity of the eluted VAN from the VAN + US + MB cylinders against planktonic* S. aureus* was significantly higher than from either the control or VAN or VAN + US specimens; and (3) in the rabbits, the activity of the eluted VAN from the VAN + US + MB cylinders against* S. aureus* was significantly higher than from either the control or VAN or VAN + US specimens. The present results suggest that VAN-loaded PMMA cement irradiated with MB-mediated US may have a role in controlling prosthetic joint infection.

## 1. Introduction

Every year, between 0.3% and 2.2% of the patients who receive primary total joint replacement (TJR) develop prosthetic joint infection (PJI), which is resistant to systemic antibiotic treatment mainly due to the low local concentration of the antibiotics [1–3]. In some of these cases, the TJR is revised, which is a costly procedure. To reduce the likelihood of occurrence of PJI, it is common to use an antibiotic-loaded poly(methyl methacrylate) (PMMA) bone cement (ALBC) to anchor the implant in the bone [4, 5]. An ALBC has the attractions of high local dose and low systemic toxicity compared to intravenously delivered antibiotics [6]. However, an ALBC has its share of shortcomings, one of which is low effectiveness against microbial species, a consequence of incomplete release of the antibiotics [6–8]. One approach that has been taken to increase the in vitro elution rate of antibiotics from an ALBC specimen is to subject it to ultrasound (US), with such increase being due to enhancement of the mass transfer rate of the antibiotics [9–15]. The ALBC combined with US seemed promising in in vitro studies but the benefits of US alone failed to achieve significant results in animal models in previous studies [10, 13–15]. Thus, without in vivo evidence, the efficacy of US exposure alone is very inconclusive for enhancing antimicrobial ability of ALBC.

Microbubbles- (MBs-) mediated US has been widely used as a method to significantly increase the elution of drugs and genetic materials [16–18]. This increase is due to the fact that MBs, which are 1–8 mm sized particles with a gas core and a stabilizing shell built up by protein, lipid, or biocompatible polymers [19], could serve as nuclei for ultrasonic cavitation and lower the energy threshold for US-induced sonoporation [16–18]. MB-mediated US was reported to significantly enhance the bactericidal ability of an antibiotic in both planktonic and biofilm forms [9, 20–22] by stimulating the passive or active uptake of antibiotics, also through sonoporation [23].

In the present work, we hypothesized that when a vancomycin- (VAN-) loaded PMMA bone cement is irradiated using MB-mediated US, each of the three parameters will be significantly greater than the corresponding value in the two other study groups. The parameters are in vitro VAN elution rate, in vitro activity of the eluted VAN against planktonic* Staphylococcus aureus* (*S. aureus*), a microorganism commonly found in PJI cases [24], and activity of eluted VAN against* S. aureus* inoculated in a periprosthetic rabbit model. The other two study groups were VAN-loaded PMMA bone cement and VAN-loaded PMMA bone cement irradiated using US. Furthermore, we determined (1) planktonic* S. aureus *concentration in a group comprising plain cement specimens (control group) and (2) the morphology and ultrastructure of planktonic* S. aureus *collected from each of the above-mentioned four study groups at the end of the in vitro activity study.

## 2. Materials and Methods

### 2.1. Elution Study of VAN-Loaded Bone Cement

Three sets of specimens were fabricated. In the first group, 2 g of VAN (Vancopin; Eli Lilly, Indianapolis, IN, USA) was blended with 40 g of the powder of a commercially available PMMA bone cement brand (CMW Endurance; DePuy, Leeds, UK), after which the blended powder mixture was manually mixed with the cement liquid (20 mL). The dough was delivered into molds to produce cylindrical test specimens, with diameter and length of 2 mm and 20 mm, respectively (VAN group). The second group comprised VAN specimens irradiated using US (VAN + US group) and, in the study group, VAN specimens were irradiated using MB-mediated US (VAN + US + MB group). In each group, the specimens (*n* = 6) were placed in a beaker containing 40 mL phosphate buffered saline (PBS), at 37°C, which was continuously stirred. The specimens in the VAN + US and VAN + US + MB groups were irradiated at the bottom of the beakers for 24 h using an US generator (Nexus; Hexin Biomedical Devices, Hangzhou, China [15]), with the conditions being as follows: transducer frequency, 1 MHz; acoustic intensity, 300 mW/cm^2^; and 3 : 10 duty cycle. In our previous work, we showed that (1) with the US intensity used there was no damage to the skin and soft tissues in an animal model [15]; and (2) bacterial reduction of VAN induced by an intermittent US pattern was markedly greater than that induced by a continuous pattern [14]. In the VAN + US + MB group, the MB contrast agent (SonoVue, Bracco, Milan, Italy) was reconstituted in 5 mL of 1 N saline, resulting in preparation that contained 2 × 10^8^–5 × 10^8^ MBs/mL [25]. According to the manufacturer's manual, stability of MB is sustained 6 h after preparation but MB might not survive for that long under US exposure. Thus, the MB supplementation was carried out by replacing 0.5 mL of solution in each beaker with fresh MB solution every 4 h. A 100 *μ*L aliquot of the supernatant was taken from each beaker at a number of time points (0, 4, 16, and 24 h). The same amount of fresh PBS was pipetted into each beaker to maintain 40 mL of PBS in each beaker after each sampling. In each group, the VAN concentration in the PBS was measured at designated time points using a fluorescence polarization immunoassay (FPIA) (AxSYM, Abbott Laboratories, Chicago, IL, US).

### 2.2. In Vitro Antimicrobial Activity Study

There were two parts in this study and, in each part, four study groups used, namely, control, VAN, VAN + US, and VAN + US + MB.

In the first part, a customized water bath apparatus was used, with four 3 cm diameter transducers fixed at the bottom. The water bath was designed to fit a polystyrene microtiter 6-well plate (Corning, Corning, NY, USA), four wells of which could be placed directly above the four ultrasonic transducers accordingly. The transducer was located 2 mm below each well, and US was transmitted through the bottom of the 6-well plate after the water bath was filled with sterile 1 N saline, whose temperature was kept at 37°C using a heating rod and monitored by a thermometer. Six-well plates containing cultivating planktonic* S. aureus* were used for each of the four study groups.

For each of the groups, the final concentration was 10^7^ CFU/mL, MB 2 × 10^7^–5 × 10^7^ MB/mL, and VAN 2 *μ*g/mL, which was the MIC of VAN against* S. aureus.* 0.5 mL of the solution in each well was replaced with fresh MB or VAN or NS every 4 h and the experiment lasted for 24 h. In the VAN + US and VAN + US + MB groups, the* S. aureus *was irradiated using the same parameter values as in the elution study.

In the second part of the study, the colony-forming growths were observed by inoculating 20 *μ*L of planktonic* S. aureus* onto sheep blood agar plates (Hardy Diagnostics, Santa Maria, CA, Canada) immediately after treatment and then culturing in 5 vol./vol.% CO_2_, at 37°C for 24 h.

Quantitative viable bacterial cultures were performed using a colony-forming units (CFU) counting method. In each part of the study, each experiment was run at least three times and the* S. aureus *concentration (bacterial density) was calculated as log (number of CFU)/mL.

### 2.3. Determination of Morphology and Ultrastructure of Bacteria

In each of the four study groups used in the in vitro antimicrobial activity study, the planktonic* S. aureus *was collected at the end of the experiments and then centrifuged at 10,000 rpm for 6 min. Then, the pellets were fixed with 1% osmium and were then left at room temperature for 1 h. After that, the samples were dehydrated and fixed in Epon 812 resin and left to polymerize for 3 days. Each sample was cut into thin slices, approximately 90 nm, and then the morphology and ultrastructure of the bacteria were observed and photographed using a transmission electron microscope (TEM, JEM-1230; JOEL, Tokyo, Japan) [26].

### 2.4. In Vivo Study

**Figure 1:**
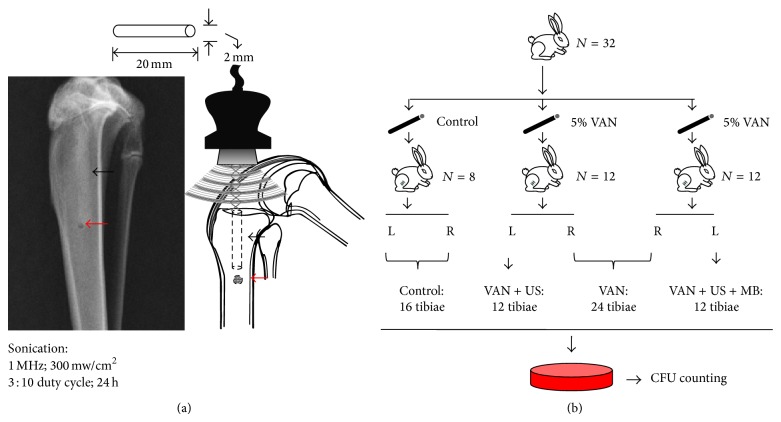
Schematic drawings of (a) the placement of the cement specimens in the tibia of a rabbit and (b) the design of the in vivo study. VAN: vancomycin; MBs: microbubbles; US: ultrasound; CFU: colony-forming units. Black arrow: bone cement; red arrow: 1-mm-diameter hole.

Thirty-two healthy adult New Zealand white rabbits (Animal Center of Zhejiang University, Hangzhou, China), with an average weight of 2.52 ± 0.48 kg, were obtained 7 days before surgery to be acclimatized to the Clinical Animal Laboratory at the Second Affiliated Hospital of Zhejiang University, Zhejiang, China. The experimental protocol was approved by the Animal Ethics Committee of Zhejiang University. Experimental periprosthetic infection was established in both the right and the left tibiae using identical procedures for both sides under sterile conditions. After anesthesia was administered, a medial parapatellar incision was made, and the patella was removed. Following the exposure of the tibia plateau, a 3 mm diameter stainless steel drill bit was used to make a 22 mm deep bone tunnel through the center of the tibial plateau into the proximal tibia canal. Subsequently, a small suction tube was inserted into the canal to remove as much blood and bone marrow as possible. Thereafter, a cement specimen was inserted with its end 2 mm above the cutting plane. Next, perforation at the proximal tibia was performed 22 mm below the tibia plateau with a 1 mm diameter hole (Figure 1(a)). 100 *μ*L of a suspension of* S. aureus *was injected into the proximal tibial canal through this hole. Then, the hole was sealed with surgical wax. A silicon drain was placed at the proximal end of the cement specimen. The muscles were rejoined, as was the skin. The animals lost 3.58 ± 1.24 mL of blood without receiving an intravenous infusion or prophylactic antibiotic postoperatively.

The 64 tibiae from these 32 rabbits were then divided into the four groups as follows: (a) control group: 8 left tibiae and 8 right tibiae; (b) VAN group: 24 right tibiae; (c) VAN + US group (12 left tibiae); and (d) VAN + US + MB group (12 left tibiae) (Figure 1(b)).

The US exposure system was set up as follows: each of the transducers was fixed onto the skin above the tibial plateau from the anterior direction. The center of the probe was located 1 cm away from the transducers. Each tibia was irradiated from 0 to 24 h after surgery.

For the VAN + US + MB tibiae, 100 *μ*L of the MB contrast agent, SonVue MB (2 × 10^8^–5 × 10^8^ MBs/mL), was injected into the 1 mm diameter hole into the tibial canal at 4 h intervals, for a total of 400 *μ*L per treatment. The animals were kept in a customized restrainer to avoid movement.

At the end of each test in a rabbit, the animal was sacrificed with an intravenous overdose of 10 vol./vol.% pentobarbital. Under sterile conditions, the tibiae of each group were excised and cleaned of tissue residue and the proximal portion was harvested by cutting off the tibia at the plane of the drilled hole. After the cement specimen was removed, bone marrow and hematoma material from the proximal portion of the tibia were thoroughly collected, placed into a sterile centrifuge tube, and weighed. Normal saline was added to the centrifuge tube to make 1 mL of suspension. Then the suspension was homogenized with a tissue grinder (Ultra-Turrax T8; IKA-Werke, GMBsH & Co., Staufen, Germany). Quantitative bacterial cultures of tibial tissue were performed using a CFU counting method. The* S. aureus *concentration (bacterial density) in the tissue was calculated as log CFU per gram of tibia tissue.

### 2.5. Statistical Analysis

All results are presented as mean ± standard (SE). Comparison of results between any two studied groups was conducted by unpaired and two-tailed nonparametric Mann-Whitney test using Prism 5 software (GraphPad Software, San Diego, CA). The value *P* < 0.05 was considered statistically significant.

## 3. Results

### 3.1. In Vitro VAN Elution and Antimicrobial Activity

**Figure 2:**
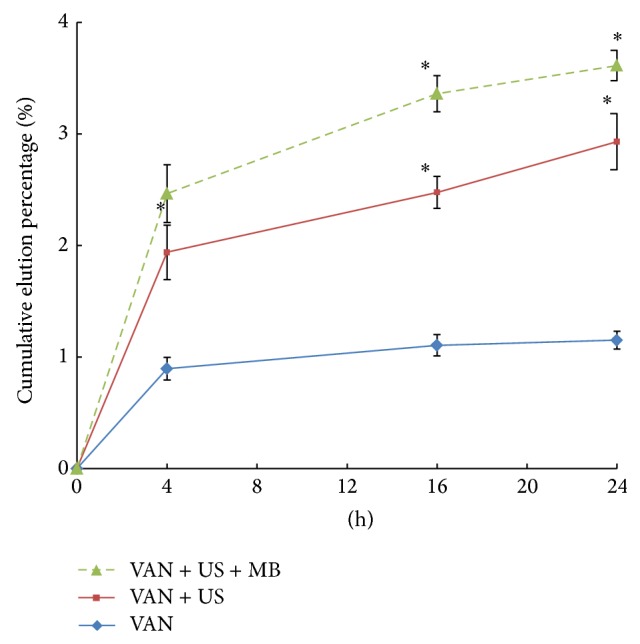
Summary of the in vitro results for VAN elution. Asterisk (∗) denotes significant difference compared to results for VAN group. VAN: vancomycin; MBs: microbubbles; US: ultrasound; CFU: colony-forming units.

At the end of the study time (24 h), the cumulative amount of VAN eluted from VAN + US + MB specimens was significantly greater than that from either VAN or VAN + US specimens (Figure 2).

**Figure 3:**
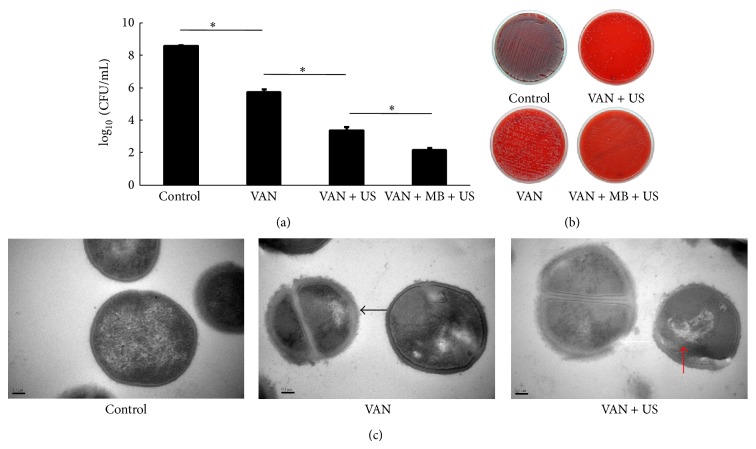
Summary of the (a) in vitro results for VAN activity against planktonic* S*.* aureus*, (b) colony-forming growth in sheep blood agar plates, and (c) the morphology of planktonic* S*.* aureus* cells. In (a), asterisk (∗) denotes significant difference. In (c), black arrow indicates increase in roughness and thickness of cell wall, white arrow indicates broken cell wall, and red arrow indicates shape-altered cell and cell debris. VAN: vancomycin; MBs: microbubbles; US: ultrasound; CFU: colony- forming units.

The eluted VAN from the VAN + US + MB group was significantly more effective against the planktonic* S. aureus* than in any of the other study groups (*P* < 0.001) (Figure 3(a)), consistent with the observations of the colony-forming growth on sheep blood agar plates (Figure 3(b)) and the morphology and ultrastructure of the bacterial species (Figure 3(c)). Specifically, the mean thickness of bacterial cell wall in the VAN + US group (62.18 ± 3.54 nm) was significantly greater than that in either the VAN group (45.51 ± 2.00 nm) (*P* < 0.001) or the control group (27.11 ± 2.11 nm) (*P* < 0.0001). In Figure 3(c), there are no bacterial cell results for the VAN + US + MB group because the planktonic* S. aureus* collected from this group could not be centrifuged due to the low concentration of the bacterial species.

### 3.2. Antibacterial Action of Eluted VAN In Vivo

**Figure 4:**
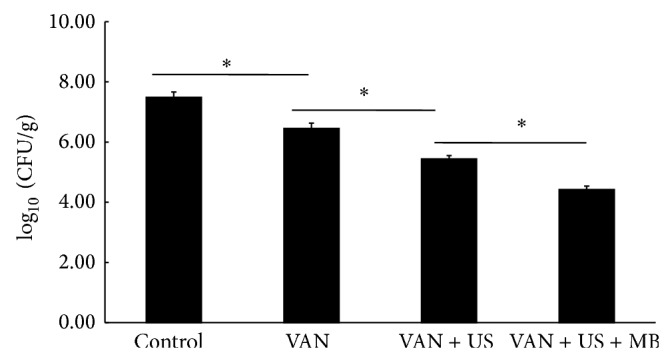
Summary of the surviving amount of the* S*.* aureus* in the rabbits. Asterisk (∗) denotes significant difference. VAN: vancomycin; MBs: microbubbles; US: ultrasound; CFU: colony-forming units.

There were no burns or other skin damage in the VAN + US or VAN + US + MB groups of rabbits. The viable count of* S. aureus* that survived when VAN + US + MB cylinders were implanted was significantly lower than in each of the other study groups (*P* < 0.0001) (Figure 4).

## 4. Discussion 

PJI is an intractable clinical condition, mainly due to the local subinhibitory concentration after systemic infusion of antibiotic(s), subsequently leading to bacterial resistance. ALBCs comprise one approach for the prevention and treatment of PJI. However, the impermeability of the PMMA to the antibiotic(s) to a large extent results in a slow and incomplete release of the antibiotic(s) and, sometimes, failure to reach minimal antibiotic concentration [6, 8]. Therefore, an effective therapeutic strategy should be developed to (1) enhance release of the antibiotic(s) from the cement and (2) potentiate the bactericidal effects of the released antibiotic(s).

Several attempts using US to serve this purpose were made by the present workers as well as others. In our previous study, we reported that favorable results using low intensity US (46.5 kHz, 100–300 mW/cm^2^) alone are inconclusive as far as the bactericidal efficacy of ALBC in vivo is concerned [15]. Consistent with our results, Ensing et al. also demonstrated only a trend that bactericidal effects of ALBC could be accelerated under 48 h insonation alone in vivo (48 kHz, 167–500 mW/cm^2^, 1 : 3 circle) [10]. MB-mediated US was considered as a promising candidate to strengthen the acoustic biophysical effects as MBs could serve as nuclei for ultrasonic cavitation and lower the energy threshold for US-induced sonoporation [16–18]. In this context, MB-mediated US was believed to increase the elution of antibiotics of ALBC and simultaneously enhance bactericidal ability of released antibiotics [9, 20–22]. Thus, the current study was designed to explore the feasibility of MB-mediated US for reducing viable* S. aureus* in vitro and to confirm its efficacy in a clinical relevant PJI model.

The present results showed a significant increase in in vitro elution rate of VAN from VAN-loaded cement cylindrical specimens subjected to insolation by MB-mediated US compared to the case when either VAN-loaded cement specimens or VAN-loaded cement specimens irradiated using US alone. Normally, the release of drug from bone cement is controlled by surface phenomena and antibiotics diffusion within ALBC, leading to a biphasic elution curve: a transient initial release peak is followed by a long-term low release [7, 8]. The potential explanation of our elution results is the stable cavitation and ultrasonic pressure wave produced by US irradiation on the surface of ALBC, accelerated rate of mass transfer from PMMA matrix to interface of implant. Additionally, the oscillation or rupture of microbubbles by US exposure remarkably increased microstream and sheer stress, which further facilitate the elution of antibiotics [8, 9, 12–15, 22].

Also, in the in vitro tests, the significantly reduced number of surviving planktonic* S. aureus *cells when VAN + US + MB specimens were used (compared to the case with each of the other three study groups) was consistent with the trend in the number of colony-forming growths of the bacterial species on sheep blood agar plates. Two influential factors mainly determining the antimicrobial efficacy of one antibiotic are the concentration of drug and the susceptibility of bacteria. In addition to the increase of drug elution, shear stress produced on the surface of bacteria by ultrasound gives rise to transient micropores in the bacterial wall through which active or passive uptake of the antibiotics is facilitated [23, 27]. With the presence of external MB, ultrasound was believed to exert additional microstream and shear stress on bacteria wall, and created more pores during sonication. One of our recent studies partly supported the above theory by showing that MB-mediated US, when combined with gentamicin, could further enhance the bactericidal effect against* Escherichia coli* (*E. coli*) partly through the destruction of the bacterial cell wall [22].* S. aureus *applied in the current study is a gram positive bacterium with much higher tensile strength of cell wall than the gram negative* E. coli* [22]. Surprisingly, the cell wall changes of* S. aureus *detected by TEM after treatment of VAN + US might prove sonoporation could even occur in planktonic gram positive bacteria and then facilitate the antimicrobial activity of antibiotics [22]. This is of great clinical merit as* S. aureus *is more common but more difficult to treat than* E. coli* in clinical orthopedic surgery.

The present results also showed that, in the tibiae of rabbits, when VAN-loaded bone cement specimens irradiated using MB-mediated US were inserted, there was significant enhancement of the action of the released VAN against planktonic* S. aureus* compared to when either plain cement, VAN, or VAN + US specimens were inserted. The primary reasons were based on the increased elution of VAN and the enhanced bacterial wall destruction, both of which are already discussed. Moreover, this is the first clinical relevant model to strongly support the feasibility of ALBC in combination with MB-mediated US for prevention of PJI. The PJI models adopted by previous studies are inserting infected disks subcutaneously on the back of animals, in which antibiotics were spread into such spacious subcutaneous areas, resulting in limited local concentration [10, 20, 25]. In contrast to this, our model well mimicked an acute established periprosthetic infection within 24 h after total keen arthroplasty and a small prosthesis-related gap would help to preserve effective concentration [10, 25]. The commercially available MB contrast agent we adopted in the current experiment (SonoVue) has been used as diagnostic aids to scan the various organs of body for so many years. Thus, it is well known to be safe and compatible even when administrated into the circulatory system of human [28]. ALBC in combination with MB-mediated US was proved here to effectively clear peri-implant planktonic bacteria before the more resistant biofilm was formed.

The study has a number of limitations. First, we used only one antibiotic loading in the bone cement (2 g of VAN mixed with 40 g of the cement powder). Second, for both insolation by US and insolation by MB-mediated US, we used only one set of process variables (frequency, acoustic intensity, duty cycle in the case of US, and volumes of MB and saline in the MB reconstitution). These may not be the optimal values of these process variables.

## 5. Conclusion

In in vitro tests, we found that when VAN- (VAN-) loaded PMMA bone cement specimens subjected to MB-mediated US, both the VAN elution rate and the bactericidal effect of the eluted antibiotic against planktonic* S. aureus *were each significantly greater compared to cases when either VAN alone or VAN + US was used. In tests on the tibiae of New Zealand white rabbits, the same trend for the bactericidal effect was found as was the case in the in vitro tests. It thus appears that VAN + US + MB cement may have a role to play in prevention of prosthetic joint infections.
